# Molecular mechanism of action and potential biomarkers of growth inhibition of synergistic combination of afatinib and dasatinib against gefitinib-resistant non-small cell lung cancer cells

**DOI:** 10.18632/oncotarget.24814

**Published:** 2018-03-27

**Authors:** Miao Wang, Alex Yuang-Chi Chang

**Affiliations:** ^1^ Department of Oncology, Johns Hopkins Singapore International Medical Center, Singapore; ^2^ Department of Oncology, Johns Hopkins University, Baltimore, MD, USA

**Keywords:** molecular mechanism, growth inhibition, synergistic combination, gefitinib resistance, non-small cell lung cancer

## Abstract

Epidermal growth factor receptor - tyrosine kinase inhibitor (EGFR-TKI) is the first choice of treatment for advanced non-small cell lung cancer (NSCLC) patients harbouring activating EGFR mutations. However, single agent usually has limited efficacy due to heterogeneous resistant mechanisms of cancer cells. Thus drug combination therapy would offer more benefits by synergistic interactions and avoidance of resistance emergence. In this study, we selected 8 NSCLC cell lines with different genetic characteristics as research models to investigate the efficacy of 4 agents (gefitinib, cetuximab, afatinib and dasatinib) and their combinations. As a single agent, both afatinib and dasatinib showed more inhibition against cell proliferation than gefitinib and cetuximab. Afatinib combined with dasatinib demonstrated significantly high efficacy against 7 gefitinib-resistant NSCLC cell lines. Moreover, it reversed the resistance to the 4 studied single agents in PTEN mutated NSCLC cells. By studying the activity of EGFR, Src and their downstream signalling pathways including PI3K/PTEN/Akt, Ras/Raf/MEK/ERK, Src/FAK and JAK/Stat, we demonstrated the synergistic interaction between afatinib and dasatinib was not only due to their blockage of different signalling pathways but also the complemental inhibition of the related signalling molecules such as Stat3. We also found that the level of Src, Stat3, and MAPK may be useful biomarkers predicating synergism between afatinib and dasatinib for the treatment of gefitinib-resistant NSCLC cells.

## INTRODUCTION

Epidermal growth factor receptor (EGFR) is over expressed in the majority of NSCLC and genetic alterations in the tyrosine kinase domain of EGFR are associated with the sensitivity to treatment with molecular targeted agents [1]. Although tyrosine kinase inhibitor (TKI) showed encouraging clinical responses in NSCLC patients harbouring EGFR mutations [2], almost all patients developed resistance to these inhibitors after initial clinical response. About half of acquired resistance was associated with secondary T790M EGFR mutation in exon 20 [3] and 21% of such resistance was linked to c-Met amplification [4]. Beside the acquired resistance, many NSCLC patients were carrying primary resistant genetic status such as EGFR wide-type, k-ras mutation and others. Approximately 2–9% of NSCLC tumours lacked PTEN, and PTEN loss has been considered indicative of primary or acquired resistance to EGFR–TKIs [5–9]. We also noticed that increased expression of Src was reported in 50% to 80% of NSCLC. In addition, high levels of Src kinase activity have been observed in NSCLC, with the degree of kinase activity correlating with tumour size [10]. In a study of 60 cancer cell lines, the NSCLC cell lines had the highest median Src activity [11]. Src family signalling plays an important role in several signalling pathways, mainly involving in Ras/Raf/MAPK, PI3K/Akt, STAT and FAK signalling pathway. More importantly Src family kinase can interact with both EGFR and MET pathways, and may result in resistance to EGFR-TKI. Hence, each type of resistance needed to be overcome by different strategies.

Cetuximab (Erbitux^**®**^, Bristol-Myers Squibb) is a chimeric monoclonal antibody against the extracellular domain of EGFR. It has been shown to provide modest survival benefit when adding to chemotherapy for advanced NSCLC, especially in patients with high expression of EGFR. However, cetuximab is not expected to work in gefitinib or erlotinib resistant EGFR mutant cases. Dasatinib (Sprycel^**®**^, Bristol-Myers Squibb; BMS-354825), a potent, multi-targeted, oral inhibitor of Src family kinases, Bcr-Abl, c-Kit, platelet-derived growth factor receptor (PDGFR) and Eph receptors, has been shown to have anti-tumour effects in solid tumours. In preclinical studies, NSCLC cells treated with dasatinib showed decreased cell growth, substrate-dependent changes in cell morphology and changes in downstream signalling leading to a reduced capability for invasion [12, 13]. In EGFR-dependent NSCLC cell lines, treatment with dasatinib results in apoptosis. In the clinical setting, initial pharmacodynamic data have demonstrated that patients with solid tumours exposed to dasatinib showed substantially inhibitory Src activity. Furthermore, no dose-limiting toxicity was observed in a dose escalation study in patients with solid tumours [14]. However, a phase II study showed that dasatinib as monotherapy in molecularly unselected NSCLC patients did not appear promising [15]. Afatinib (Gilotrif, Boehringer Ingelheim, BIBW2992) is a novel, orally bio-available, anilinoquinazoline compound, developed and designed as an irreversible dual inhibitor covalently bound to Cys 773 of EGFR and Cys 805 of HER2 [16]. Afatinib was shown to be superior to chemotherapy, pemetrexate and cisplatin as the first line treatment for patients with advanced NSCLC with activating EGFR mutations, but it was disappointing that the drug was unable to improve OS compared with placebo, in a population of patients with prior exposure to reversible EGFR TKIs, despite the evidence of significant clinical activity such as superior response rates, time to progression as compared to placebo [17]. The response rate to afatinib alone was reported to be 7%. Osimertinib, the third generation of EGFR TKI specifically designed against EGFR activating mutation with resistant T790M mutation, was reported to have higher response rates and longer PFS than chemotherapy in these group of patients, but again majority of patients developed resistant mutation in EGFR genes and by other mechanisms [18]. All of above, the described agents alone showed limited efficacy in clinic due to development of resistance. Therefore, a new strategy to combat EGFR–TKI resistance caused by various mechanisms is needed.

Drug combination may offer more benefits in the treatment of advanced NSCLC from synergistic interactions and avoidance of resistance emergence. A previous study indicated that dasatinib in combination with gefitinib achieved more inhibition of cell growth than that of either dasatinib or gefitinib alone [12]. Another study showed that combined EGFR targeting with afatinib and cetuximab achieved encouraging clinical response rate of 30% in NSCLC patients with acquired resistance (AR) to prior erlotinib or gefitinib [17]. Most recently, combination of osimertinib and gefitinib was demonstrated some efficacy in patients with C797S and T790M EGFR-mutated NSCLC, but the improvement is brief [19]; the activation of Src family kinases and focal adhesion kinase (FAK) was revealed to contribute to the resistance to afatinib, erlotinib and osimertinib in afatinib-resistant HCC827 cells, which lost the amplified, mutated EGFR genes [20]. Osimernitib combined with dasatinib was reported to overcome the resistance to first generation EGFR-TKI, gefitinib for example, in NSCLC patients with acquired T790M [21]. Up to date, there are limited reports on the efficacy of combination of EGFR-TKI and dasatinib overcoming the resistance to EGFR-TKI in NSCLC with various genetic characters.

In this study, we defined the efficacy of afatinib combined with dasatinib in gefitinib resistant NSCLC cells harbouring different genetic mutation characteristics and determined the potential biomarkers predicating synergism of the combination. Moreover, this is, to the best of our knowledge, the first report revealed the growth inhibition by combination of afatinib and dasatinib against PENT mutations in NSCLC cells, which demonstrated strong resistance to either afatinib or dasatinib alone, and we further investigated and uncovered the molecular mechanism of tumour inhibition for reversing the drug resistance.

## RESULTS

### Cell growth inhibition by single agent varied against 8 NSCLC cell lines

**Figure 1:**
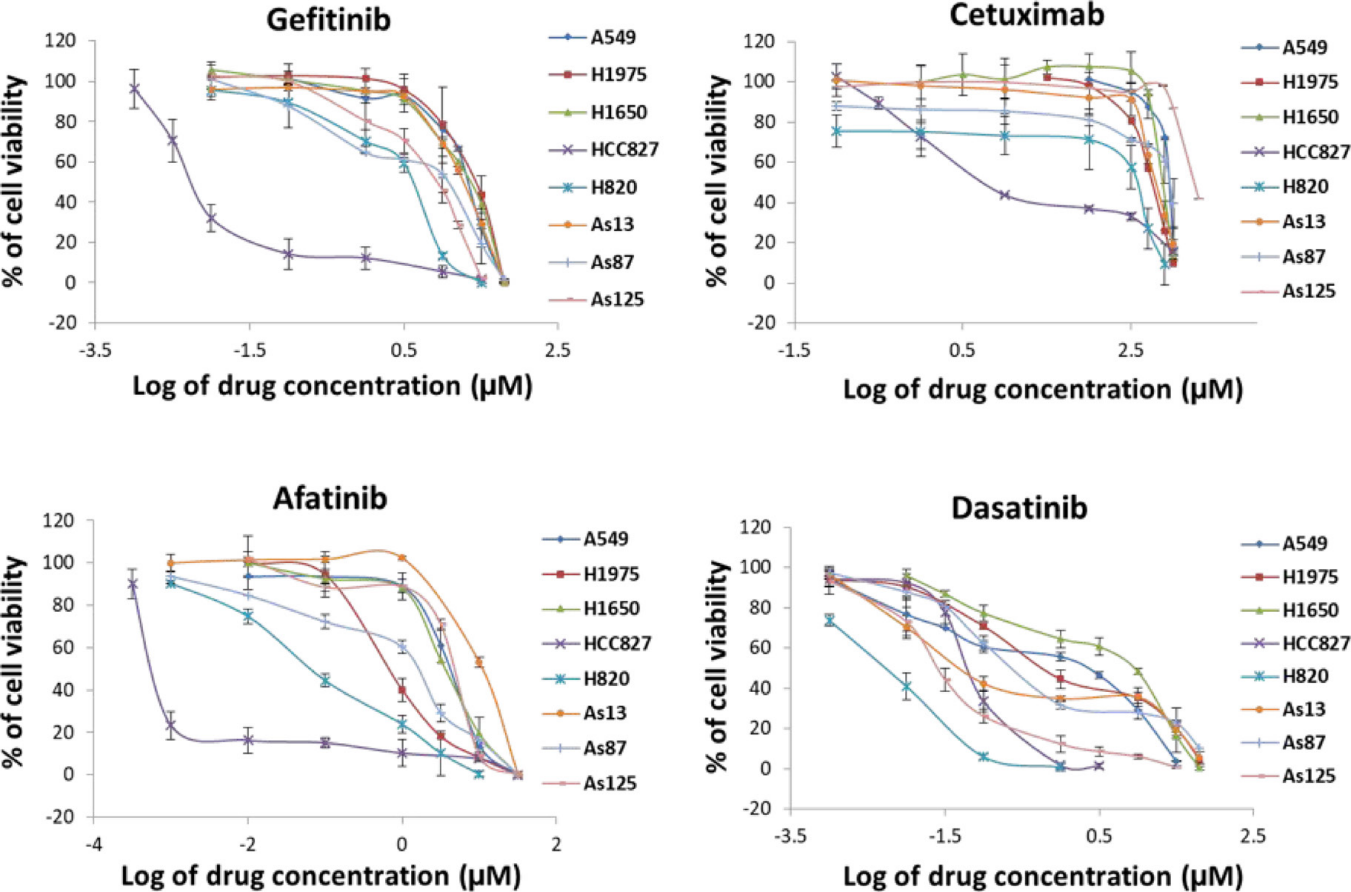
Comparison of growth inhibition of NSCLC cells by 4 different agents 8 NSCLC cell lines were treated by gefitinib, cetuximab, afatinib, and dasatinib alone, respectively. Individual % of cell viability is the mean ± SD from at least three experiments.

We used 5 commercial NSCLC cell lines (A549, H1975, H1650, HCC827, and H820), and 3 cell lines (As13, As87, and As125) which were established by us from NSCLC patients’ pleural effusion. Their mutation status were shown in Table 1. HCC827 carrying exon19 deletion of EGFR, is a gefitinib- sensitive cell line [4]. We used it as a positive control. Inhibition of tumour cell proliferation by single agent was tested by MTS assay (Figure 1) and IC50 of each drug in the studied cell lines was calculated (Table 1). HCC827 was sensitive to all of the four agents (gefitinib, cetuximab, afatinib and dasatinib) with the IC50 value from 0.7 nM to 50 nM. The other 7 cell lines showed resistance to both gefitinib (IC50: 4.4 ∼ 25.5 μM) and cetuximab (IC50: 2.4 ∼ 12 μM). H1975 carrying L858R + T790M EGFR mutation is considered as acquired resistant to gefitinib. We noted afatinib showed more inhibition against T790M mutation than gefitinib. This was mainly because afatinib irreversibly bound to Cys773 of EGFR to overcome the mechanism of resistance to gefitinib (16). H820, harbouring exon19 deletion (E746-E749) + T790M + MET amplification, also showed positive response to afatinib. Since MET amplification is able to activate HER3-dependent PI3K/Akt pathway and its occurrence is independent of T790M [4], and afatinib has proven activity of preventing phosphorylation of HER3 [16], the growth inhibition of H820 may be caused by blocking the activity of EGFR, HER2 and HER3 and their downstream signalling pathway. Afatinib was unable to reverse the resistance to gefitinib in A549, H1650, As13, As87, and As125 cell lines. In the other hand, 5 gefitinib resistant cell lines (H1975, H820, As13, As87, and As125) showed good response to dasatinib. Indicating that Src play an important role in cell proliferation of NSCLC cells. Both H1650 and A549 were resistance to all the four agents. H1650 carries exon19 deletion (delE746-A750) + deletion of exon 9 of PTEN. Loss of PTEN expression in EGFR-mutant cells correlates with decreased sensitivity to EGFR-TKI [8]. A549 harbours WT-EGFR and HER2, activating K-ras G12s point mutation. Patients with K-ras mutations have poor sensitivity to EGFR TKI [22, 23] and unfavourable prognosis [24, 25]. Overall, the efficacy of dasatinib against NSCLC cells *in vitro* is significantly stronger than gefitinib (*p* < 0.001) and cetuximab (*p* < 0.05), and no significant difference was found between dasatinib and afatinib.

**Table 1:** Comparison of sensitivities to 4 molecular target drugs in 8 NSCLC cell lines carrying various genetic status

IC50 Cell lines	Gefitinib (μM)	Dasatinib (μM)	Afatinib (μM)	Cetuximab (μM)	Genetic background of cell lines
A549	21.5 ± 1.8	2.2 ± 0.05	4.5 ± 0.9	5.7 ± 0.06	WT-EGFR and HER2, activating K-ras G12s point mutation
H1975	25.5 ± 7.7	0.8 ± 0.1	0.8 ± 0.09	3.7 ± 0.16	L858R + T790M double substitution
H1650	22.5 ± 1.2	8.8 ± 1.1	3.8 ± 0.4	5.1 ± 0.09	exon19 deletion (delE746-A750) + deletion of exon9 of PTEN
HCC827	0.008 ± 0.004	0.07 ± 0.006	0.0007 ± 0.00007	0.05 ± 0.004	exon19 deletion
H820	4.4 ± 0.9	0.06 ± 0.005	0.6 ± 0.1	2.4 ± 0.4	exon19 deletion (E746-E749) + T790M + MET amplification
AS13	18.3 ± 1.5	0.08 ± 0.005	11.2 ± 0.9	4.2 ± 0.62	L858R + missense mutation (R776C) in exon20, WT-k-ras
AS87	12.3 ± 5.1	0.6 ± 0.08	1.7 ± 0.2	6 ± 0.8	WT-EGFR, WT-k-ras
AS125	8.8 ± 1.7	0.04 ± 0.02	5.4 ± 0.2	12 ± 0.4	WT-EGFR, WT-k-ras

Individual IC50 values (μM) were the mean **±** SD calculated from at least 3 experiments.

### Afatinib combined with dasatinib increased inhibition of NSCLC cell proliferation

**Figure 2:**
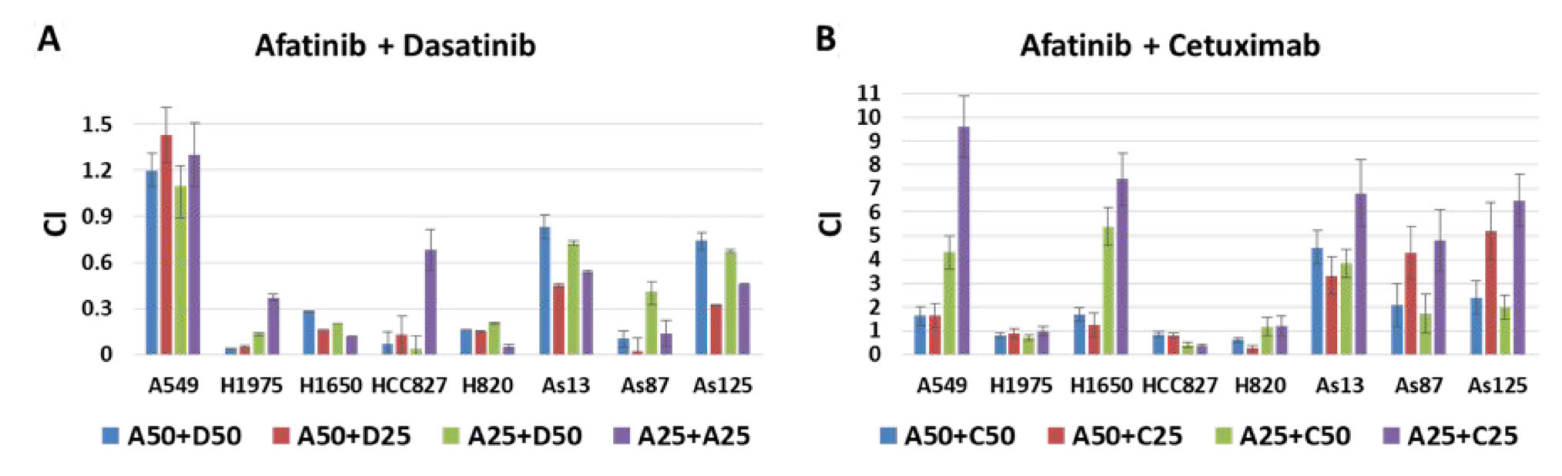
Combination effect of afatinib combined with either dasatinib or cetuximab in 8 NSCLC cell lines (**A**) Drug interaction between afatinib and dasatinib at 4 different concentration combinations, for example, A50 + D50 indicated the combination of afatinib and dasatinib at the dosage of IC50 when treated the NSCLC cells alone. (**B**) Drug interaction between afatinib and cetuximab at 4 different concentration combinations, for example, A50 + C25 indicated the combination of afatinib and cetuximab at the dosage of IC50 and IC25 when treated the NSCLC cells alone, respectively. CI < 0.9, indicating the synergistic interaction between 2 drugs. Individual CI is the mean ± SD from at least 3 experiments.

Drug combination index (CI) was calculated for each two drug combination at the designated dosage. Combination of afatinib with dasatinib achieved significant growth inhibition against all the studied cell lines except A549 (Figure 2A). Synergistic interaction between afatinib and cetuximab was only observed in HCC827, H1975 and H820 cell lines, which were sensitive to afatinib (Figure 2B). The synergy between afatinib and dasatinib was significantly stronger than that of afatinib and cetuximab (*p* < 0.001).

### Identification of potential biomarkers predicating the synergism between afatinib and dasatinib

**Figure 3:**
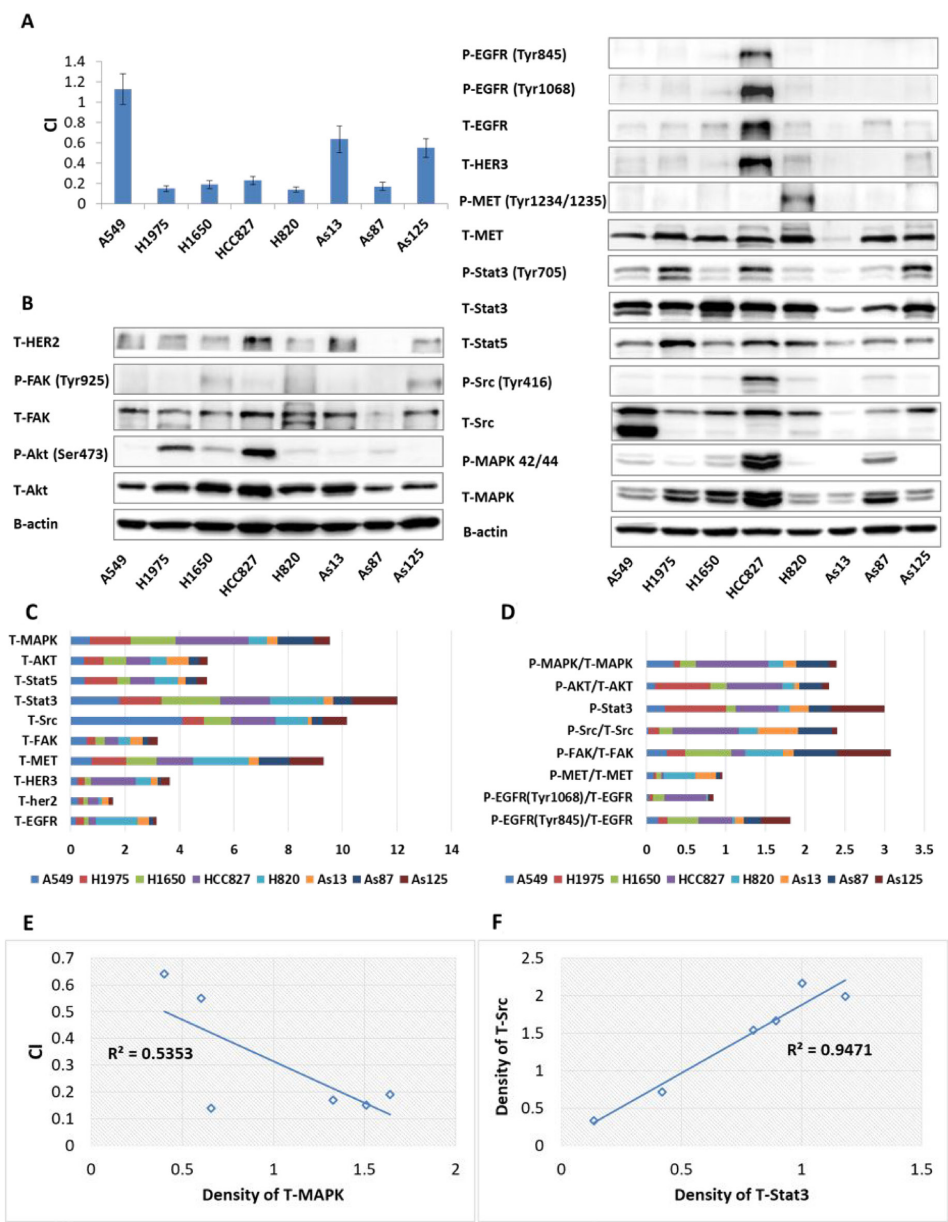
Baseline proteins expressions as well as combination index (CI) in NSCLC cells (**A**) CI indicated the interaction between afatinib and dasatinib in 8 NSCLC cell lines. (**B**) Baseline expression of receptor tyrosine kinases and downstream signaling molecules determined by western blot, β-actin was used as the loading control. (**C**) The expression ratio of the studied proteins to β-actin quantified by ImageJ software. (**D**) The expression ratio of phosphorylated protein to total protein quantified by ImageJ software. (**E**) Significant correlation between the synergistic interaction of afatinib plus dasatinib and baseline expression of MAPK (*p* < 0.05). The Pearson correlation coefficient (r) was equal to 0.733. (**F**) Significant correlation between baseline expression level of Src and Stat3 (*p* < 0.001). The Pearson correlation coefficient (r) was equal to 0.972. The *p*-value correlation probability from r was calculated by student’s *t* test. Results represented the mean ± SD from at least three experiments.

In order to identify potential biomarkers predicating the synergetic effects between afatinib and dasatinib, we measured the expression level of total (T) proteins and phosphorylated (P) proteins in the signalling pathways which may be affected by afatinib or dasatinib (Figure 3A–3D). Strong synergism between afatinib and dasatinib was correlated with high expression level of T-MAPK (*p* < 0.05) (Figure 3E) in 6 gefitinib-resistant cell lines which positively responded to the combination of afatinib and dasatinib. We also found that baseline expression level of T-Src significantly correlated with T-Stat3 (*p* < 0.001) (Figure 3F). These findings might imply the synergistic interaction between afatinib and dasatinib on the signaling pathways affected by Src, Stat3 and MAPK.

### Afatinib combined with dasatinib inhibits the activity of EGFR, HER2, Src and downstream signaling in H1650 cells

**Figure 4:**
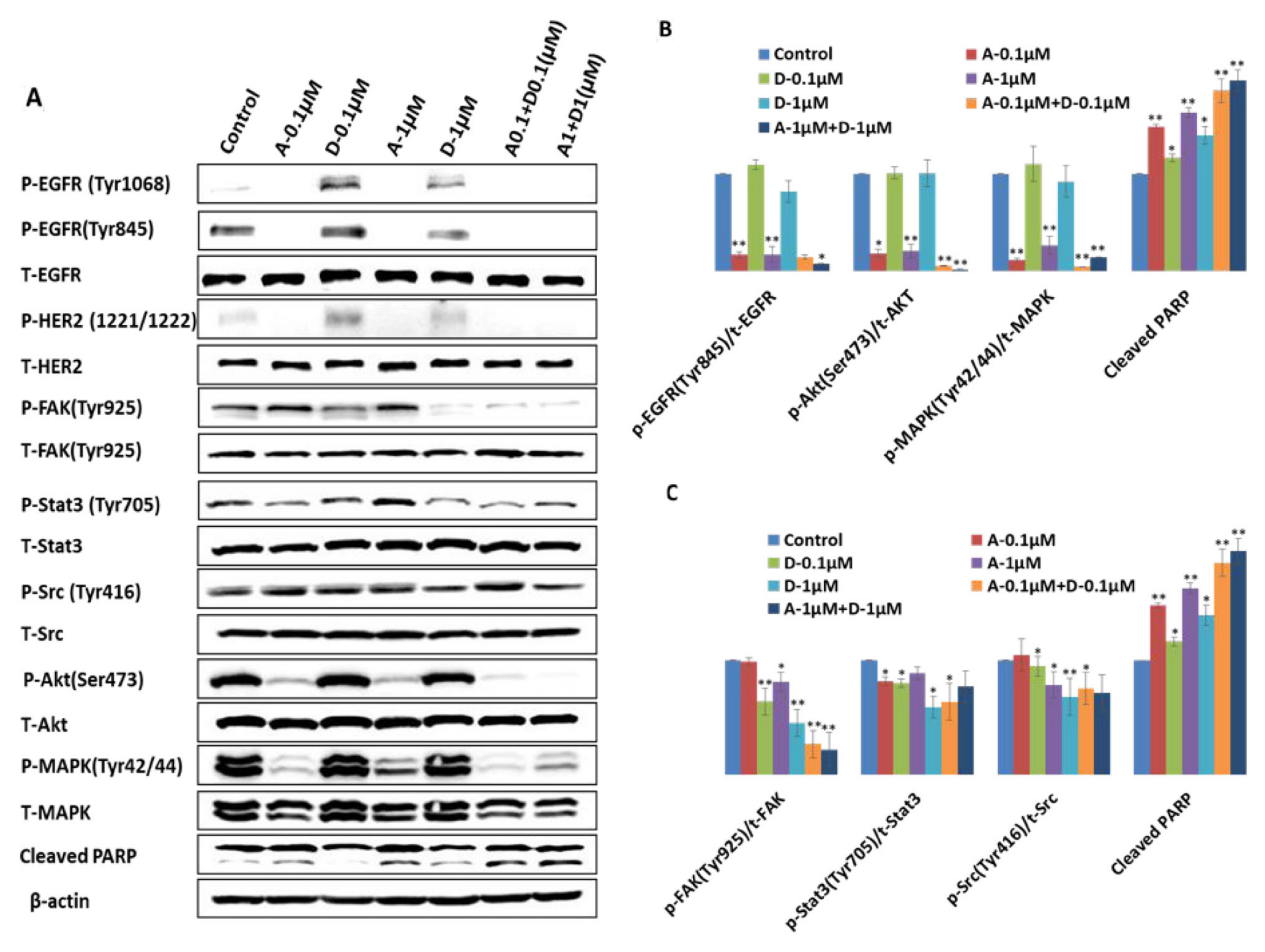
Different effect of afatinib, dasatinib and their combination on the targeted proteins and downstream molecules in H1650 cells (**A**) Western blot analysis showed different expression level of the targeted proteins and downstream molecules upon the designated treatments. For example, A-0.1 µM indicates the treatment of afatinib at the dosage of 0.1 µM, and A0.1 + D0.1 (µM) means the combination of 0.1µM of afatinib and 0.1 µM of dasatinib. (**B**, **C**) Quantification of the ratio of phosphorylated protein to total protein by ImageJ software. The results were mean ± SD obtained from at least three experiments. *P*-value was calculated by student’s *t* test (^**^p < 0.01; ^*^p < 0.05).

In order to study the mechanism underlying synergetic tumor inhibition by combination of afatinib and dasatinib, H1650 cells were treated by afatinib, dasatinib and their combination at the designated doses. The targeted proteins were analyzed by western blotting and the ratio of P-protein to T-protein was calculated by ImageJ software (Figure 4A–4C). Phosphorylation of EGFR at Tyr845 (P-EGFR845) was completely inhibited by afatinib alone at the dosage of 0.1 μM (*p* < 0.01), slightly decreased by dasatinib (1 μM) alone, and the complete inhibition was observed by the combinations (*p* < 0.05). The baseline expression level of both P-EGFR (Tyr1068) and P-HER2 (Tyr1221/1222) was very weak. Their phosphorylation was completely abolished by afatinib alone and the combinations (*p* < 0.01), even though it was slightly increased by dasatinib alone. Src activity (P-Src) was inhibited by dasatinib at the dosage of 1 μM (*p* < 0.05) but not afatinib. The combination of afatinib (1 μM) and dasatinib (1 μM) demonstrate the inhibition of P- Src (*p* < 0.05). However, there was no further inhibition was observed by the combination comparing with the treatment by dasatinib alone. There was a dose-dependent inhibition of phosphorylation of FAK at tyr925 by dasatinib, and the inhibition level at both of the dosage 0.1 μM and 1 μM were significant (*p* < 0.01), enhanced inhibition by the combination was clearly demonstrated (*p* < 0.01).The activity of Akt (P-Akt) was reduced by afatinib alone (*p* < 0.01), and further deduction was observed by the combinations (*p* < 0.05). Afatinib also showed a strong inhibition on activating of MAPK. 0.1 μM of afatinib significantly inhibited P-MAPK (Tyr42/44) (*p* < 0.01), which wasn’t affected by dasatinib. Furthermore, 0.1 μM of afatinib combined with 0.1 μM of dasatinib enhanced afatinib’s inhibition effect on phosphorylation of MAPK (*p* < 0.05). Afatinib showed 15% of inhibition on P-Stat3 (Try705) at the dosage of 0.1 μM (*p* < 0.05), and no significant inhibition was shown at the dosage of 1 μM; dasatinib demonstrated more reduction of the activity of Stat3 at both 0.1 μM (23%) and 1 μM (48%), respectively (*p* < 0.01) when compared with the control; the combination of afatinib and dasatinib at the lower concentration (afatinib: 0.1 μM, dasatinib:0.1 μM) induced more inhibition of the activity of Stat3 than either afatinib or dasatinib alone (*p* < 0.01). The increasing of cleaved PARP was observed by each individual drug treatment (*p* < 0.05), and further increasing was induced by the combinations (*p* < 0.01). The activated HER3, MET, PTEN, and Stat5 were not detected in H1650 cells.

### Afatinib in combination with dasatinib enhanced apoptosis of H1650 cells

**Figure 5:**
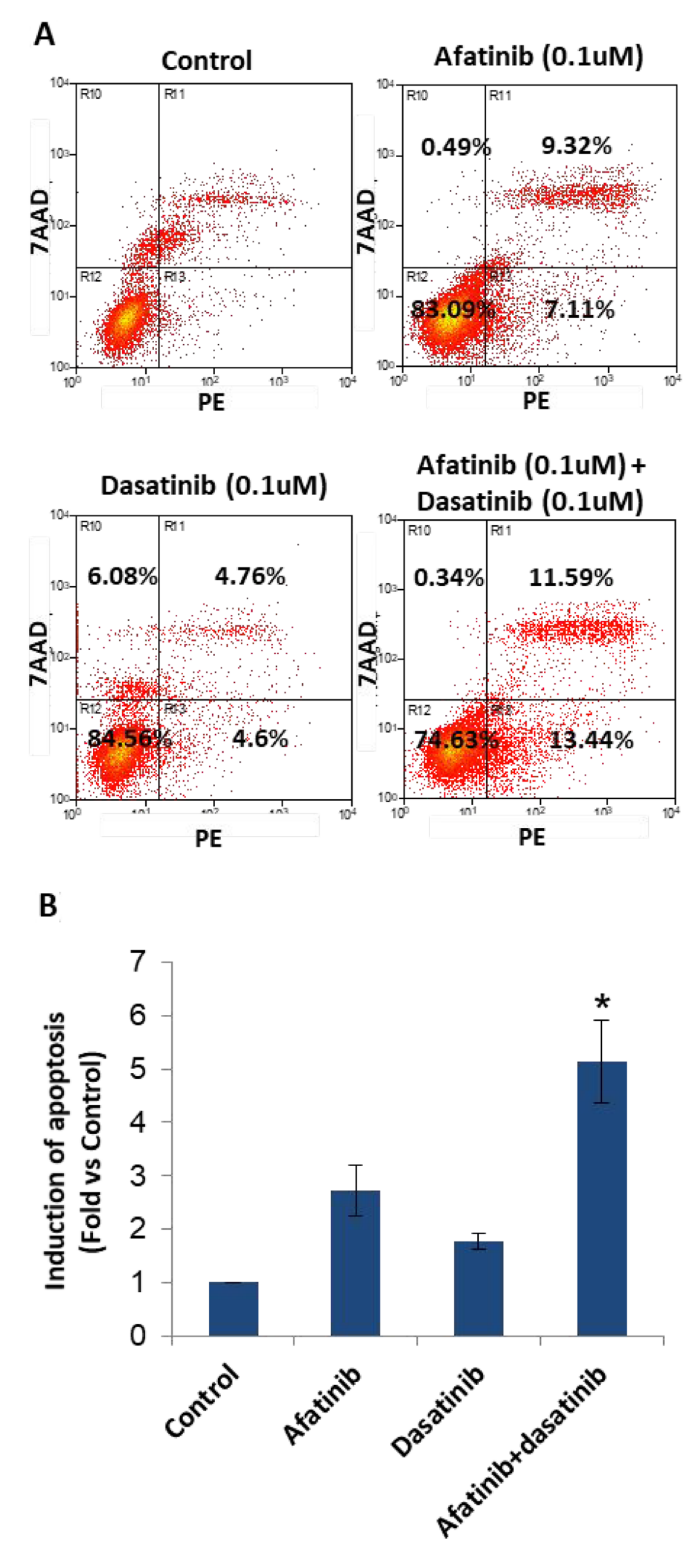
Induction of apoptosis by the treatment of afatinib, dasatinib, and their combination in H1650 cells (**A**) Flow cytometry analysis showed the induction of apoptosis upon the designated treatment. (**B**) Statistic chart showed the significant induction of apoptosis by the combination of afatinib and dasatinib comparing with afatinib or dasatinib alone. The results were mean ± SD obtained from at least three experiments. *P*-value was calculated by student’s *t* test (^*^p < 0.05).

H1650 cells were treated as described in methods. Early stage of cell apoptosis was induced (Figure 5), they were 2.61% (control), 7.11% (0.1 μM of afatinib), 4.6% (0.1 μM of dasatinib) and 13.44% (afatinib + dasatinib). Afatinib combined with dasatinib significantly enhanced apoptosis of H1650 cells (*p* < 0.05).

### Effects of combined afatinib and dasatinib on cell adhesion, migration and invasion of H1650

**Figure 6:**
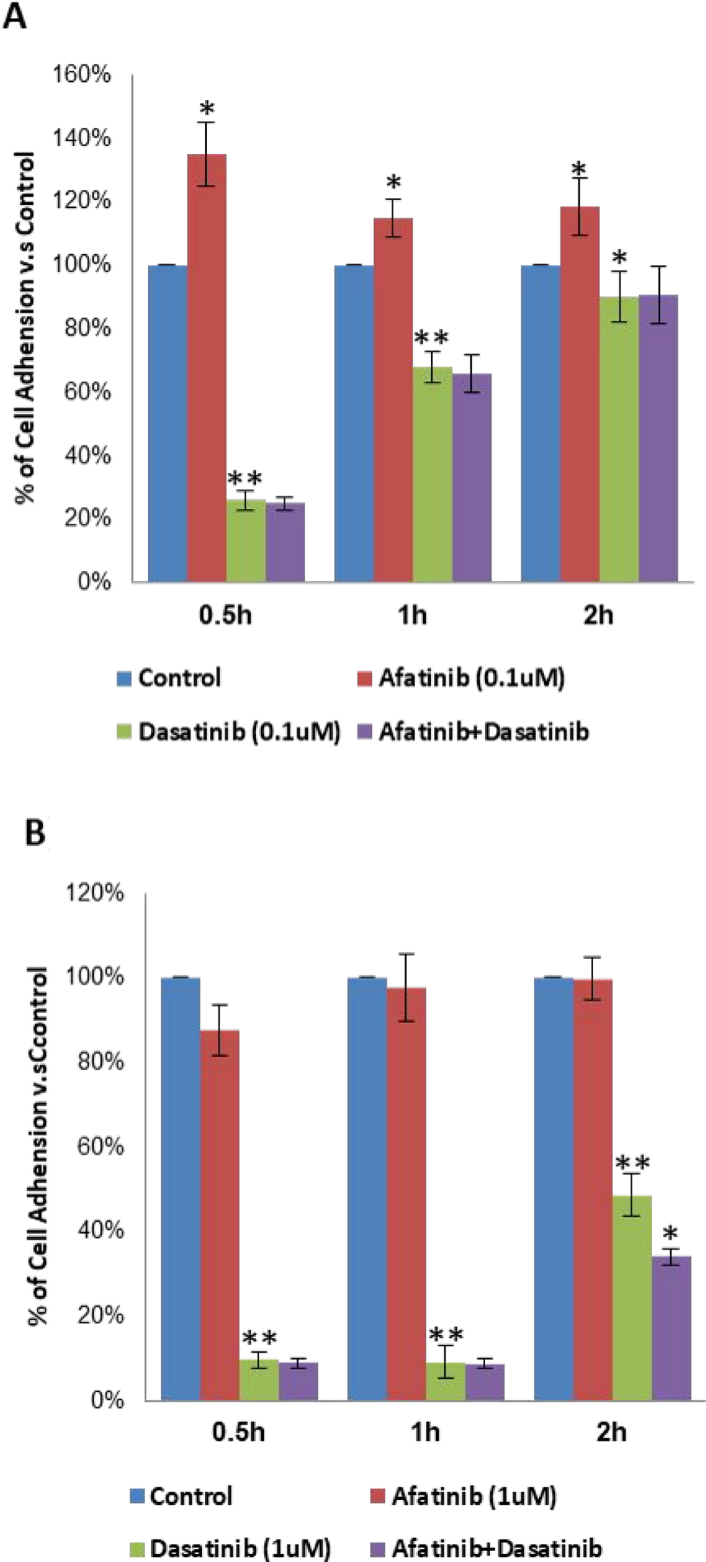
Inhibition of cell adhesion to collagen I by the treatment of afatinib, dasatinib, and their combination in H1650 cells (**A**, **B**) % of cell adhesion to collagen I upon the designated treatment at different dosage. The results were mean ± SD obtained from at least three experiments. *P*-value was calculated by student’s *t* test (^**^p < 0.01; ^*^p < 0.05).

In general, both afatinib and dasatinib showed dose dependent inhibition of cell adhesion to collagen I. Particularly, 0.1μM of dasatinib significantly reduced cell adhesion by 74%, 44% and 10% after 0.5 h (*p* < 0.01), 1 h (*p* < 0.01) and 2 h (*p* < 0.05) treatments, respectively. In contrast, 0.1 μM of afatinib increased H1650 cells adhere to collagen I after 0.5 h, 1 h and 2 h (*p* < 0.05), the combination didn’t show more effect than dasatinib alone (75%, 32%, 9% respectively), but it reversed the negative effect of afatinib (Figure 6A). With increasing the dosage to 1 μM, dasatinib further inhibited cell adhesion by 90%, 91% and 51% respectively at above time points, afatinib didn’t show any significant effect (12%, 2%, 0%) on cell adhesion; more inhibition by the combination of afatinib and dasatinib (66%) was observed on cell adhesion after 2 h (*p* < 0.05), but no significant difference was found after 0.5 h and 1 h (Figure 6B).

**Figure 7:**
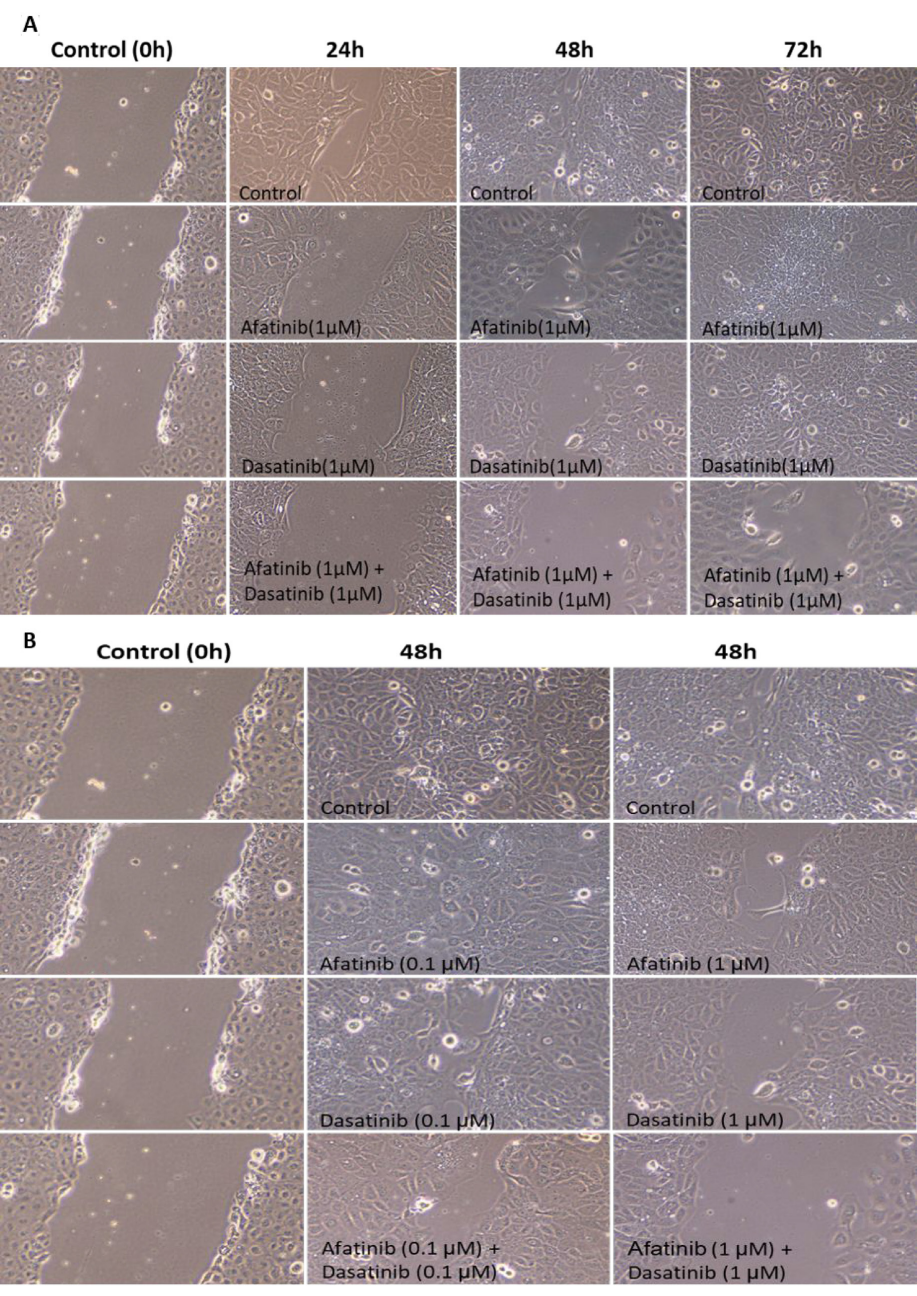
Time and dose-dependent inhibition of cell migration by the treatment of afatinib, dasatinib, and their combination in H1650 cells (**A**) Enhanced inhibition of cell migration at various time duration by the combination of afatinib and dasatinib. (**B**) enhanced dose-dependent inhibition of cell migration by the combination of afatinib and dasatinib.

An enhanced inhibition on cell migration of H1650 was shown at various time duration when treated by either afatinib, dasatinib alone or their combination (Figure 7A). Either afatinib or dasatinib alone significantly reduced cell migration to the wound area after 24 h and 48 h (*p* < 0.05), and the wound completely healed after 72 h. Afatinib combined with dasatinib demonstrated further deduction of cell migration after 24 h, 48 h, and 72 h comparing with each individual drug treatment alone (*p* < 0.01). Figure 7B showed the dose-dependent inhibition of cell migration in H1650 cells by afatinib, dasatinib alone as well as the combination. More deduction of the cell migration was induced by the treatment at higher dosage (1 μM) than that of the lower one (0.1 μM). There is no invasion observed in H1650 cells.

## DISCUSSION

Both afatinib and dasatinib achieved better efficacy against cell proliferation than reversible EGFR-TKI and EGFR monoclonal antibody. However, neither afatinib nor dasatinib as a single agent would meet the demand of treatment for NSCLC in clinical practice due to resistance. Afatinib combined with dasatinib showed more synergistic effect than combined with cetuximab. Theoretically, the afatinib and dasatinib combination inhibited cell signalling in different signal pathways due to synergic interaction of Src with EGFR or their downstream factors, but the gefitinib or afatinib and cetuximab only affected the same EGFR pathway [26]. Afatinib in combination with dasatinib was unable to overcome the primary resistance with K-ras mutation since the activation of mutant K-ras is non-EGFR or Src dependent [27]. However, afatinib combined with dasatinib synergistically reduced cell proliferation of H1650 which was also resistant to either afatinib or dasatinib alone. In this study, we specifically focused on this cell line to elucidate the mechanism underlying synergistic anti-cancer effect between afatinib and dasatinib.

In H1650 cells, the synergistic anti-tumour activity of the combination of afatinib and dasatinib was exhibited in cell proliferation, survival, apoptosis, adhesion and migration through the interaction amongst the various signalling pathways. Previous studies have reported that depletion of PTEN protein or loss of PTEN function reversely increases PI3K/Akt activity and causes drug resistance in a variety of cell types [28–31]. Afatinib alone completely inhibited P-EGFR (Tyr1068), P-EGFR (Tyr845) and P-HER2 (1221/1222) subsequently decreased the activity of Akt and MAPK but not complete inhibition (Figure 4B), thus suggesting that afatinib alone was not sufficient to reverse the resistance to gefitinib via blocking both EGFR/PI3k/Akt and EGFR/Ras/Raf/MAPK signalling pathways, even though it showed more efficacy than gefitinib (IC50: 3.8 µM vs 22.5 µM). In contrast, dasatinib alone didn’t have any inhibition on phosphorylation of Akt, MAPK, but slightly increased P-EGFR at the point of Tyr1068, indicating that there was no suppression effect on EGFR/PI3k/Akt and EGFR/Ras/Raf/MAPK signalling pathways but dasatinib alone reduced Src activity and subsequently inhibited P-EGFR (Tyr845), thus H1650 cells could survive via the activated EGFR/PI3K/Akt / EGFR/Ras/Raf/MAPK pathway to bypass Src signalling pathway when treated by dasatinib alone. The interaction between Src and FAK has been shown to active both cell motility and invasion [32, 33]. Upon stimulation of EGF or PDGF receptors, FAK auto-phosphorylated, creating a high affinity binding site for Src, the association between Src and FAK resulted in activation of Src and phosphorylation of FAK at various tyrosine sites. The Src/FAK complex phosphorylated a number of other focal adhesion proteins and activated other intra-cellular signalling pathway including PI3K/PTEN/Akt, and Ras/Raf/MEK/ERK signalling pathways [34]. In this study, we found that dasatinib alone substantially inhibited the activity of FAK at the site of Tyr925, which enable dasatinib to produce more inhibition of cell adhesion and migration than afatinib. The inactivation of EGFR by afatinib could further inhibit the formation of FAK/Src complex, resulted in down-regulation of the activity of FAK. As a result, we observed: 1) enhanced the inhibition on both cell adhesion and migration (Figures 6 and 7); 2) reduced the binding of PI3K to FAK, and sequentially fully abolished the phosphorylation of Akt (Figure 4); 3) inhibited the binding of Gr2 to FAK at the Tyr925 and significantly reduced the activity of MAPK (Figure 4). In addition, Src-mediated pathway is also involved in activating signal transduction and transcription (Stat3) to mediate cell cycle progression and against apoptosis [35]. We found that dasatinib produced significantly more inhibition of P-Stat3 (Tyr705) than afatinib. Dasatinib might restrain the activity of Stat3 to reduce the cell survival by inactivation of Src, and it demonstrated more efficacy on Stat3 signalling pathway than that of afatinib. Previous studies found that MAPK played a direct inhibitory modification on Stat3 in tumour cells [35]. When the activity of MAPK was restricted by afatinib, Stat3 might be activated relevantly to inhibit cell apoptosis and increase cell survival. From our results, dasatinib was able to overcome this inhibitory modification by inhibiting the activity of Stat3. The small dosage of afatinib (0.1 μM) achieved more inhibition of P-MAPK than that of 1 μM. This was in accordance with that combination between small dosages (0.1 μM of afatinib + 0.1 μM of dasatinib) was more synergic than that of large dosages (1 μM of afatinib + 1 μM of dasatinib). The large dosage may trigger the inhibitory modification among the related cell signalling pathways to bypass the inhibition. We also found that afatinib demonstrated more efficacy on the cell apoptosis (Figure 5), which complemented and confirmed by the cleavage of PARP. The increased cleaved PARP by the combination of afatinib and dasatinib was further confirmed the enhanced induction of apoptosis.

According to our results, afatinib inhibited cell proliferation and induced apoptosis via affecting PI3K/PTEN/Akt, and Ras/Raf/MEK/ERK signalling pathways; dasatinib decreased cell survival, adhesion and migration, and induced apoptosis through affecting Src/FAK and JAK/Stat signalling pathways. The synergistic interaction between afatinib and dasatinib was not only due to their blockage of different signalling pathways but also by inhibition of the cross-talking among the related signalling molecules. T. Yoshida has demonstrated that afatinib combined with dasatinib produced significant *in vivo* tumour regression in PC9GR xenograft studies [36]. Clinical phase I trial on this combination is ongoing to discover its efficacy by Lee Moffitt Cancer Center (https://clinicaltrials.gov identifier: NCT01999985). JAK/Stat signalling pathway may play important role in the gefitinib resistant cells. So far there is no effective therapy for NSCLC patients with PTEN deletions. Our study suggests combination of afatinib and dasatinib may be effective in this particular group of patients. In our study, we didn’t test osimertinib either alone or combination with dasatinib. This should be an area of subsequent investigation in view the superior efficacy of osimertinib over erlotinib/gefitinib as the first line treatment of EGFR sensitive mutation NSCLC with less toxicities [18].

In conclusion, combination of afatinib and dasatinib had more synergistic effect than cetuximab plus afatinib against gefitinib resistant NSCLC cells. The combination reversed the resistance to EGFR-TKI-resistant H1650 cells with PTEN mutations via affecting SFK/FAK, PI3K/PTEN/Akt, Ras/Raf/MEK/ERK, and JAK/Stat pathways. The level of MAPK, Src and Stat3 may be useful biomarkers predicating synergism between afatinib and dasatinib for the treatment of gefitinib-resistant NSCLC cells. Our work also provided strong scientific support for future *in vivo* study as well as clinical trials.

## MATERIALS AND METHODS

### Drugs

Cetuximab (monoclonal antibody against EGFR) was purchased from Merck AG and stored at 4° C. Gefitinib (EGFR TKI) was purchased from Biaffin GmbH & Co KG (proteinkinase.de, Germany). Afatinib (irreversible EGFR and HER2 kinase inhibitor) was bought from LC Laboratories, USA. Dasatinib (Src kinase inhibitor) was obtained from Bristol-Myer Squibb Company. Stock solution was prepared at 10 mM in pure DMSO and stored in aliquots at −80° C. These agents were further diluted with culture medium immediately before use.

### Cell lines

A549, H1975, H1650, HCC827, and H820 were obtained from American type Culture Collections (ATCC). As13, As87, and As125 NSCLC cell lines were derived from patients’ pleural effusion by us. All cell lines were cultured in RPMI culture medium with Hepes and L-glutamin (PAA laboratories cell Culture Products, Austria), containing 10% Fatal Bovine Serum (Invitrogen, USA), 1% antibiotic with 100 UI/ml Penicillin and 100ug/ml Streptomycin (Invitrogen, USA). Incubation condition was set at 37° C in a humidified atmosphere of 95% air and 5% CO_2_. The culture medium was changed 2 to 3 times a week and cells were subcultured using trypsin/EDTA (Invitrogen, USA).

Direct sequencing was applied for mutational analysis of the EGFR, PTEN and K-Ras genes for the three self-established cell lines. Exons 18∼21 of EGFR, exon 2 of *K-*ras, and all 9 exons of PTEN were first amplified using intron-based primers (two PCR reactions for exon 8 of PTEN). The amplicons were then purified for sequencing analysis on ABI PRISM 3100 genetic analyzer (Applied Biosystems) [37].

### Growth inhibition assay

CellTiter 96 Aqueous Non-Radioaction cell proliferation Assay Kit (Promega Corporation, USA) was used for growth viability assays. 3000–8000 cells from 8 cell lines were plated in 96-well flat-bottomed plates and cultured for 24 hours (h). Cells were exposed to serially diluted drugs in completed RPMI cell culture medium for an additional 72 hours. 20 μl MTS/PMS solution was added into each well containing 100 μl of the culture medium. Then, the cells were incubated for 3 h at 37° C before measurement of absorbance at 490 nm with a Benchmark Plus microplate spectrophotometer (Bio-RAD, USA). Absorbance values were expressed as a percentage of that for untreated cells, and the concentration of dasatinib resulting in 50% growth inhibition (IC_50_) was calculated for each cell line. A sensitive cell line to test agents is arbitrarily defined if IC_50_ is less than or equal to 1 μM or falls below the C*max* of each agent on the basis of clinical phase I pharmacokinetic data [38].

### *In-vitro* drug combination analysis

*In-vitro* drug combination efficacy was analysed following the method of the previous study [38]. Briefly, according to the cytotoxicity effect of single drug in NSCLC cell lines, IC_25_ and IC_50_ were calculated respectively. Two different drugs were combined in the following sequence: IC_25_ of first drug was combined with IC_25_ and IC_50_ of the second drug, similarly, IC_50_ of the first drug was also combined with IC_25_ and IC_50_ of the second drug. The NSCLC cells were treated with the combination concurrently for 72 hours. The cell growth inhibition assay was done by MTS assay. The combination index (CI) between two different drugs was evaluated by the method of Chou and Talalay. CI less than 0.90 indicates a synergistic interaction; CI between 0.90 and 1.10 indicates additive, and CI >1.10 indicates an antagonism effect.

### Treatments by afatinib, dasatinib and the combination

Cells were plated at 5 × 10^5^ per well of 6-well plates in completed RPMI culture medium for overnight. After additional 24h serum starvation cells were treated with various concentrations of afatinib, dasatinib and their combination for 3 hours prior to 100 ng/ml EGF stimulation, and 30 minutes later the cells were used for immunoblotting, cell adhesion, migration, invasion and apoptosis assay.

### Western blotting analysis

The cells were lysed for protein extraction using 1× RIPA protein extraction buffer with protease inhibitor and phosphatase inhibitor (Thermo scientific, Pierce Biotechnology, USA). The total protein concentration was measured by BCA kit (Pierce Biotechnology, USA). Isolated proteins (30 μg/lane) were separated by 8% SDS-PAGE and transferred to a nitrocellulose membrane by the iblot device (Invitrogen Corporation, CA). The membranes were blocked with 5% BSA at room temperature for 1 h and then subjected to immunoblots using primary antibodies at 4° C overnight, followed by incubation with secondary goat anti-rabbit IgG conjugated to horseradish peroxidase for 1 h at room temperature. Labelled protein was visualized by chemiluminescence (Immobilon, Millipore Corporation, USA) and captured, using β-actin and β-tubulin expression as the internal standard.

### Cell apoptosis assay

Cells were detached by 3 mM EDTA and washed with 1x PBS, then stained with Annexin V and 7-AAD (BD), and analysed by flow cytometry (Cyan, ADP, Beckman) for detection of apoptosis cells.

### Cell adhesion assay

1.5 × 10^5^ of pre-treated cells were seeded in collagen I coated 96-well plate and incubated for 0.5 h, 1 h, 2 h and 4 h respectively. Cells adhered to collagen I were fixed and stained with cell stain solution (Chemicon International, USA), after washing stained cells were incubated with 100 µl of extraction buffer (Chemicon International, USA), and then optical density [16] was measured at 570 nm by Benchmark Plus Micro-plate Spectrophotometer (Bio-RAD, USA).

### Cell migration assay

Cells were treated following the procedure described in “Treatments by afatinib, dasatinib and the combination”. Confluent monolayer of cells were scraped with a fine 200 µl pipette tip to produce a wound. The medium containing various concentrations of drugs were replaced by completed RPMI culture medium and incubated for another 24 h, 48 h and 72 h. Migration into the wound was captured at ×10 magnification on an invert light microscope (Olympus, Japan). Three representative areas were scored and the area moved was calculated using ImageJ analysis software.

### Cell invasion assay

Cell invasion assay was processed by using the cell invasion assay kit (Chemicon International, USA). A 24-well tissue culture plate with cell culture inserts which contained an 8 μm pore size polycarbonate membrane was used. 1.5 × 10^5^ testing cells in serum free RPMI were seeded into ECM coated insert, then RPMI with 10% FBS was placed in the 24-well plate as chemo attractants. After 72 h incubation, the cells were removed from the inner surface of the insert using a cotton-tipped swab. The cells that invaded through the ECM layer and clung to the bottom of the polycarbonate membrane were fixed and stained. The number of migrating cells per insert was captured microscopically.

### Statistical analysis

All the experiments were repeated at least 3 times. Data are reported as means ± SD, correlation coefficient (r) was calculated by the Pearson product-moment correlation coefficient, and statistical significance (*p*-value) was analysed using student’s *t* test.
